# Roles of cuproptosis-related gene DLAT in various cancers: a bioinformatic analysis and preliminary verification on pro-survival autophagy

**DOI:** 10.7717/peerj.15019

**Published:** 2023-03-17

**Authors:** Qinjing Yang, Shuangshuang Zeng, Wei Liu

**Affiliations:** 1 Department of Pharmacy, The Third Xiangya Hospital, Central South University, Changsha, China; 2 Nursing Department, The Third Xiangya Hospital, Central South University, Changsha, China; 3 Department of Pharmacy, Xiangya Hospital, Central South University, Changsha, China

**Keywords:** DLAT, Pan-cancer, LIHC, Autophagy, Immune infiltration

## Abstract

**Background:**

Studies have shown that the expressions and working mechanisms of Dihydrolipoamide S-acetyltransferase (DLAT) in different cancers vary. It is necessary to analyze the expressions and regulatory roles of DLAT in tumors systematically.

**Methods:**

Online public-platform literature on the relationships between DLAT expression levels and tumor prognosis, methylation status, genetic alteration, drug sensitivity, and immune infiltration has been reviewed. The literature includes such documents as The Cancer Genome Atlas (TCGA), Human Protein Atlas (HPA), Tumor Immune Estimation Resource 2.0 (TIMER2.0), Gene Expression Profiling Interactive Analysis 2 (GEPIA2) and Receiver Operating Characteristic plotter (ROC plotter). The molecular mechanisms of DLAT were explored with the Gene Set Enrichment Analysis (GSEA). The relationship between down-regulated DLAT and autophagy in two liver hepatocellular carcinoma (LIHC) cell lines was confirmed with the western blot method, colony formation assay, and transmission electron microscopy. Tissue microarrays were validated through the immunohistochemical staining of DLAT.

**Results:**

DLAT is upregulated in the LIHC, lung adenocarcinoma (LUAD), lung squamous cell carcinoma (LUSC), and stomach adenocarcinoma (STAD) tumors but is down-regulated in the head and neck squamous cell carcinoma (HNSC) and kidney renal clear cell carcinoma (KIRC) tumors in comparison with normal tissues. For LIHC patients treated with 5-Fluorouracil and Lenvatinib, the DLAT levels of those in the drug-resistant group are significantly high. In LIHC cells, autophagy will be inhibited, and cell death will be induced when DLAT breaks down. Moreover, there exist positive correlations between DLAT expression levels and infiltration of B cells, DC cells, Tregs, and CD8+ T cells in kidney chromophobe (KICH), breast invasive carcinoma (BRCA), prostate adenocarcinoma (PRAD), LIHC and HPV+ HNSC. In LIHC, markers of Tregs are positively correlated with DLAT. Compared with those of normal tissues, the staining intensity of DLAT and the amount of Tregs marker CD49d in LIHC increase.

**Conclusions:**

Through this study, the expressions of DLAT in various cancer types can be understood comprehensively. It suggests that DLAT may be a prognostic marker for LIHC, LUAD, LUSC, STAD and KIRC. A high DLAT expression in LIHC may promote tumorigenesis by stimulating autophagy and inhibiting anti-tumor immunity.

## Introduction

Mitochondrial pyruvate dehydrogenase complex (PDC) catalyzes the irreversible decarboxylation of pyruvate, a product of glycolysis, to produce acetyl coenzyme A and enter the tricarboxylic acid cycle, thus playing a crucial role in cellular catabolic glucose pathway (Stacpoole, 2017). The Warburg effect confirms that the marker of tumor metabolism is aerobic glycolysis rather than the mitochondrial oxidation of pyruvate (Bencze et al., 2021; Wang, Wang & Tang, 2021; Wen et al., 2021). Studies have suggested that mitochondrial function is weakened by the inhibition of PDC, causing metabolic changes in tumor cells. Thus, more attention has been attracted to exploring the roles of PDC in tumors and its therapeutic potential (Saunier, Benelli & Bortoli, 2016). Dihydrolipoamide S-acetyltransferase (DLAT), a protein presenting in the inner mitochondrial membrane, is the E2 component of PDC. The main function of DLAT is to participate in the process of mitochondrial glucose catabolism and transform the acetyl produced by the oxidative decarboxylation of pyruvate to coenzyme A (Ganetzky, McCormick & Falk, 2021).

Relationships between DLAT and tumors have been studied. These studies show that the expressions and functions of DLAT in different tumors vary. It is found that DLAT is upregulated in gastric cancer cells, and the breaking down of DLAT brings about an increase in cellular pyruvate and a decrease in cell proliferation by almost 20–45% (Goh et al., 2015). Furthermore, some bioinformatics analyses have proposed that seven glycolysis genes, including DLAT, can affect the prognosis of patients with colon adenocarcinoma and be used to construct a survival prediction model (Chen et al., 2020). For patients with prostate cancer, a natural compound, alternol, plays a specific-cancer curing role by curbing the activity of four Krebs cycle enzymes, including DLAT, contributing to the reduction of mitochondrial respiration and excessive adenosine triphosphate production in cancer cells and xenografts (Li et al., 2019). The above-mentioned effects of DLAT in mitochondrial glucose metabolism and tumor growth show that DLAT could be relevant to tumorigenesis and tumor development.

However, the roles and mechanisms of DLAT in various cancers have not yet been fully revealed. On the basis of extensive epigenomic, genomic, proteomic, and transcriptomic data on different tumors published on several public platforms, the pan-cancer analysis can identify the common characteristics and heterogeneity of specific molecules in different cancers (Liu et al., 2021). A pan-cancer analysis can comprehensively identify the expressions and alteration profiles of molecules in different types of tumors, and evaluate the relationships between clinical prognosis and prospective molecular mechanisms, thus presenting the prominent guiding significance for the clinical treatment of tumors (Cui et al., 2020). Therefore, through the reference to multiple public platforms, we introduce a pan-cancer analysis method to investigate the gene expression, DNA methylation, survival prognosis, genetic alteration, related molecular pathway, and immune infiltration of DLAT. With this pan-cancer analysis method, we aim to obtain the profiles of DLAT in various tumors and preliminarily explore the relationships and regulatory mechanisms of DLAT with tumorigenesis and clinical prognosis.

Liver hepatocellular carcinoma (LIHC) ranks fifth in incidence and fourth in the fatality of cancer worldwide (Global Burden of Disease Cancer Collaboration, 2019). Among the drug therapies for LIHC, traditional chemotherapy does not have superior efficacy. In recent years, multi-tyrosine kinase inhibitors represented by Sorafenib, Lenvatinib, and Regorafenib have been widely used to treat advanced LIHC, with certain control effects on tumor angiogenesis and tumor progression (Kudo et al., 2018; Llovet et al., 2008). Recently, the application of immune checkpoint inhibitors (ICI) in the clinical treatment of advanced LIHC has also made outstanding achievements. The combination of ICIs and targeted medicine has become a new primary treatment standard, and a large number of clinical studies related to ICIs are also underway (Giraud et al., 2021).

Based on the pan-cancer analysis, we have further discussed the potential of DLAT in predicting the prognosis and drug sensitivity of LIHC, as well as its potential mechanism and immune function. Then, we preliminarily verified some results with experiments, thus providing guidance for overcoming chemotherapy resistance and seeking immunotherapy for LIHC.

## Material and Methods

### Gene and protein expressions of DLAT

Two distinct analysis tools, which are the Xiantao tool (https://www.xiantao.love/) (Chu et al., 2022) and The University of Alabama at Birmingham Cancer data analysis Portal (UALCAN, http://ualcan.path.uab.edu/index.html) (Zhang et al., 2022b), were used to differentiate the DLAT expressions of tumors and the corresponding normal tissues in The Cancer Genome Atlas (TCGA) database. Because some tumors in TCGA lacked data on corresponding normal tissues, we referred to the Gene Expression Profiling Interactive Analysis 2 (GEPIA2) website. Then, we obtained these normal data with the log2FC cutoff value set as 0.25 in the analysis (http://gepia2.cancer-pku.cn/#analysis) (Li et al., 2021). With the UALCAN tool, we also compared the expression levels of DLAT in different tumor grades. Furthermore, we used the immunohistochemistry (IHC) staining images on the Human Protein Atlas (HPA) website to present the expression levels of DLAT in tumors and normal tissues (http://www.proteinatlas.org/) (Uhlen et al., 2015).

### Cell cultures and DLAT siRNA breakdown

Two cell lines of LIHC, Huh7, and HepG2 were provided by the Cancer Research Institute of Central South University. The cells were cultured in a DMEM medium (HyClone, Logan, UT, USA), in which penicillin-streptomycin (1%; Gibco, Waltham, MA, USA) and fetal bovine serum (10%; Gibco, Waltham, MA, USA) were added to facilitate cell growth. The incubator conditions for culturing these two cell lines were set at 37 °C with 5% CO2. Two small interfering RNAs (siRNA) were specifically synthesized to target DLAT (siDLAT-1, 5′-GGCCAACCGAAGTAACAGA-3′, and siDLAT-2, 5′-CACTCTGTATCATTGTAGA-3′; RiboBio, Guangdong, China), with a scrambled siRNA as a negative control (RiboBio, Guangdong, China). Transfection for cell lines was carried out with the Lipofectamine L3000-015 reagent (Invitrogen, Waltham, MA, USA) following the protocol of Invitrogen.

### Western blot

Using the RIPA buffer, we made lysates of two LIHC cell lines with phosphatase-protease inhibitors (Bimake, Houston, TX, USA). The BCA protein assay (Thermo Fisher, Waltham, MA, USA) was performed to calculate the protein concentrations. Protein lysates with the same volume were transferred into PVDF membranes of 0.22 µm and 0.45 µm (Millipore, USA) after exposure to SDS-PAGE electrophoresis. PVDF membranes were sealed with 5% skimmed dry milk for 1 h and then incubated with 1:1000 primary antibodies of DLAT (Signal Antibody, Greenbelt, MD, USA), LC3 I/II (CST, Danvers, MA, USA), Beclin-1 (CST, Danvers, MA, USA) and GAPDH (Proteintech, Wuhan, China) at 4 °C overnight. Immobilon Western chemiluminescent WBKLS0500 reagent (Millipore, USA) was used to present proteins on PVDF membranes.

### Colony formation assay

The test protocol has been described in our earlier studies (Chen et al., 2021b) that cells were re-suspended and seeded in 6-well petri dishes with a density of about a thousand cells in each well. After at least 14 days of incubation, supernatants of the petri dish were discarded, and clones were fixed with pure methanol for 15 min and stained with crystal violet for 20 min.

### Transmission electron microscopy (TEM)

The cells were digested with trypsin and then treated overnight with 2.5% glutaraldehyde reagent. Subsequently, the cells were transferred to the TEM Laboratory of Xiangya Hospital, China, and treated as follows. The cells were cleaned three times with an interval of 10 min in the Millonig phosphate buffer and then incubated with 1% osmium tetroxide for 1 h before being rewashed. Cells were dehydrated successively with 50%, 70%, and 90% acetone and then dehydrated twice with 100% acetone. The samples were soaked and embedded for 12 h in a mixture of acetone and resin (1:1) and then polymerized overnight with pure resin at 37 °C. After that, the samples were further incubated at 60 °C for 12 h and finally sliced into 50–100 nm ultra-thin cell sections with an ultramicrotome. The cell sections were observed under an HT-7700 transmission electron microscope (Hitachi, Japan) after being stained with uranyl acetate and lead nitrate.

### DNA methylation and genetic alteration analysis

The UALCAN tool was applied to differentiate the DNA methylation of DLAT in tumors and normal tissues in the TCGA database. With the cbioportal tool, an online genetic alteration analysis of DLAT genes in the TCGA Pan-Cancer Atlas was carried out, with the frequency of genetic alteration provided by the “Cancer Types Summary” module and the “mutation” module on the genetic mutation site (https://www.cbioportal.org/) (Gao et al., 2013). Moreover, the survival rates of cancer patients in the DLAT-altered group and the DLAT-unaltered group in the TCGA database were compared in the “Comparison” module.

### Survival prognosis analysis and function enrichment analysis

The survival maps for overall survival (OS) across all TCGA cancer types were analyzed with the GEPIA2 tool. Using the Kaplan–Meier Plotter website, we further validated the relationships between DLAT expression and multiple prognostic markers in five tumors, including breast, liver, lung, ovarian, and gastric cancers (https://kmplot.com/analysis/). In addition, with the Xiantao tool, the univariate and multivariate Cox regression analyses of DLAT mRNA levels were performed on OS endpoints of 33 different tumor types in the TCGA with all available clinical variables adjusted.

For LIHC, the “Survival analysis” module of the Biomarker Exploration of Solid Tumors (BEST) method (https://rookieutopia.com/) was applied to perform the Cox regression and Kaplan–Meier analyses on DLAT expressions. With the UALCAN tool, the Kaplan–Meier analysis on the TCGA data set revealed the impact of DLAT expression level on the LIHC patients’ survival rate. A prognosis analysis was also conducted with the Kaplan–Meier Plotter on sub-types of liver cancers with statistically significant clinical variables. In addition, the Gene Set Enrichment Analysis (GSEA) of DLAT-related genes was conducted with the Xiantao website to identify whether DLAT was involved in autophagy.

### Expressions of DLAT and responses to chemotherapy

We applied the Receiver Operating Characteristic plotter (ROC plotter) website (https://www.rocplot.org/cells) (Tibor Fekete & Gyorffy, 2022) to verify the relationships between DLAT levels and responses to chemotherapy among LIHC patients. These patients were divided into sensitive and resistant groups according to their responses to chemotherapy drug treatment on the basis of lower and upper tertiles of AUDRC.

### Immune infiltration analysis

With the “Immune” section on the Tumor Immune Estimation Resource 2.0 (TIMER2.0) website, the relationships between DLAT expressions in TCGA tumors and the infiltration of various immune cells, including B cells, DC cells, NK cells, CD8+ T cells, cancer-associated fibroblasts, macrophages, monocytes, and neutrophils can be revealed. Several algorithms were used to estimate immune infiltration, including TIMER, XCELL, QUANTISEQ, MCPCOUNTER, EPIC, TIDE, CIBERSORT, and CIBERSORT-ABS. Furthermore, an analysis of the correlations between the DLAT’s expression level and typical phenotype markers of Tregs was performed with the Xiantao tool and GEPIA2 tool. We used the “Immunotherapy” module of the BEST method to evaluate the correlations between different expressions of DLAT and the response and prognosis of immunotherapy.

### Tissue microarrays and immunohistochemistry

We used tissue microarrays of 75 pairs of LIHCs and their adjacent normal tissues (Hliv-HCC150CS-02; Outdo Biotech, Shanghai, China) approved by the Ethics Committee of Shanghai OUTDO Company (YB M-05-02). The antibodies against DLAT and CD49d (19676-1-AP, Proteintech) were used for IHC. The concentrations of the antibodies against DLAT and CD49d were 1:100. Two professional pathologists scored the staining of DLAT and CD49d in tissue microarrays separately. According to the standard intensity scoring, a score of 0 indicates a negative intensity, a score of 1 indicates a weak intensity, a score of 2 indicates a moderate one, and a score of 3 indicates a strong one. The extent scores are divided into five grades, with 0, 1, 2, 3, and 4 indicating the extent of no more than 10%, 11%–25%, 26%–50%, 51%–75%, and more than 75%, respectively. Finally, the multiplying results of intensity scores and extent scores were viewed as the staining scores that were recorded.

### Statistical analysis

All experiments were repeated three times independently. Also, the Student’s *t*-test method was used to compare the differences between these two groups’ sample data. The statistical analysis results on the online database were shown in the figures, with some analysis methods not clearly marked. Data processing was conducted with GraphPad Prism 8 and SPSS 23.0. All statistical calculation results were judged according to the following criteria: * *P* < 0.05; ** *P* < 0.01; *** *P* < 0.001.

## Results

### DLAT pan-cancer expressions

**Figure 1:**
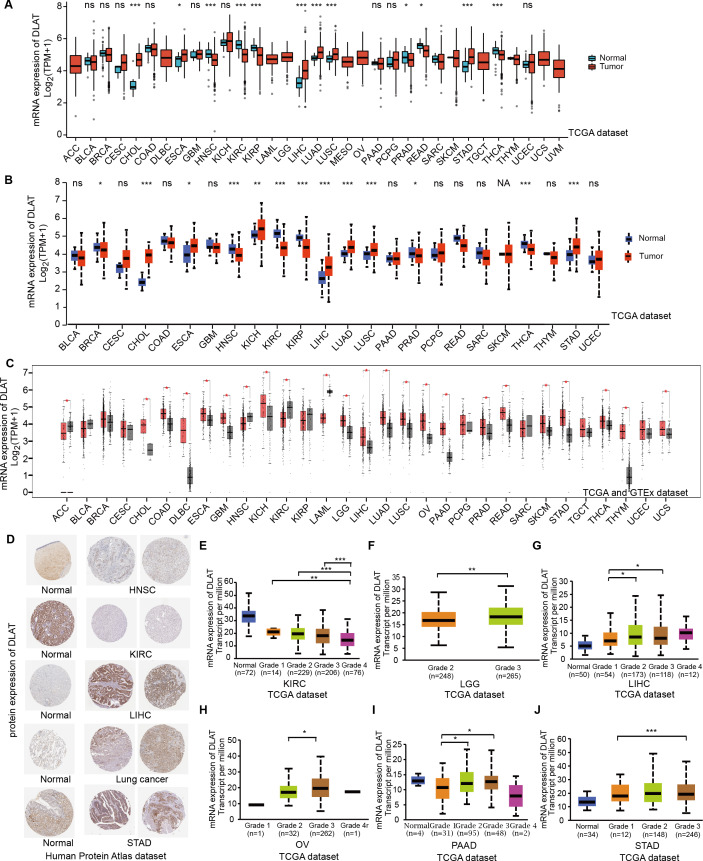
Gene and protein level of DLAT in different tumors. (A) Expression level of DLAT across TCGA tumors compared with adjacent normal tissues which analyzed by Xiantao. (B) Expression level of DLAT across TCGA tumors compared with adjacent normal tissue which analyzed by UALCAN. (C) Expression level of DLAT in tumors compared with adjacent normal tissue from TCGA and GTEx datasets which analyzed by GEPIA2. Red asterisks for significant differences. (D) IHC staining of DLAT IHC staining across normal and tumor tissues was analyzed using HPA tools in HNSC, KIRC, LIHC, Lung cancer and STAD. (E–J) Box plots to show expression levels of DLAT in KIRC, LGG, LIHC, OV, PAAD and STAD by pathological grades *P < 0.05; **P < 0.01; ***P < 0.001; ns, no significant difference; NA, missing value.

In order to compare the mRNA expression levels of DLAT in tumors and normal tissues, the Xiantao tool, UALCAN tool, and GEPIA2 tool were used to perform differential analysis on the TCGA and GTEx databases. Figs. 1A–1C show the analysis results with different databases and tools that the mRNA levels of DLAT were all significantly upregulated in certain tumor tissues, including cholangiocarcinoma (CHOL), esophageal carcinoma (ESCA), LIHC, lung adenocarcinoma (LUAD), lung squamous cell carcinoma (LUSC) and stomach adenocarcinoma (STAD). However, the mRNA levels of DLAT were all significantly down-regulated in head and neck squamous cell carcinoma (HNSC) and kidney renal clear cell carcinoma (KIRC). The IHC staining images in the HPA database confirm that the above eight tumor types that show significantly different mRNA expressions of DLAT also present different protein expressions of DLAT. The tumor tissues of LIHC, STAD, and lung cancers have stronger DLAT staining than their corresponding normal tissues, consistent with the mRNA expression results. However, as for CHOL and ESCA, there lack the IHC results.

The normal kidney, head, and neck tissues show strong or moderate DLAT staining. In contrast, their corresponding tumor tissues show weak staining (Fig. 1D). The relationship between DLAT expression and tumor grade was investigated with the UALCAN, with the result that DLAT expressions varied with the tumor grades in several tumors, including KIRC, brain lower grade glioma (LGG), LIHC, ovarian serous cystadenocarcinoma (OV), pancreatic adenocarcinoma (PAAD) and STAD (Figs. 1E–1J).

### The expressions of DLAT are related to DNA methylation and genetic alteration

**Figure 2:**
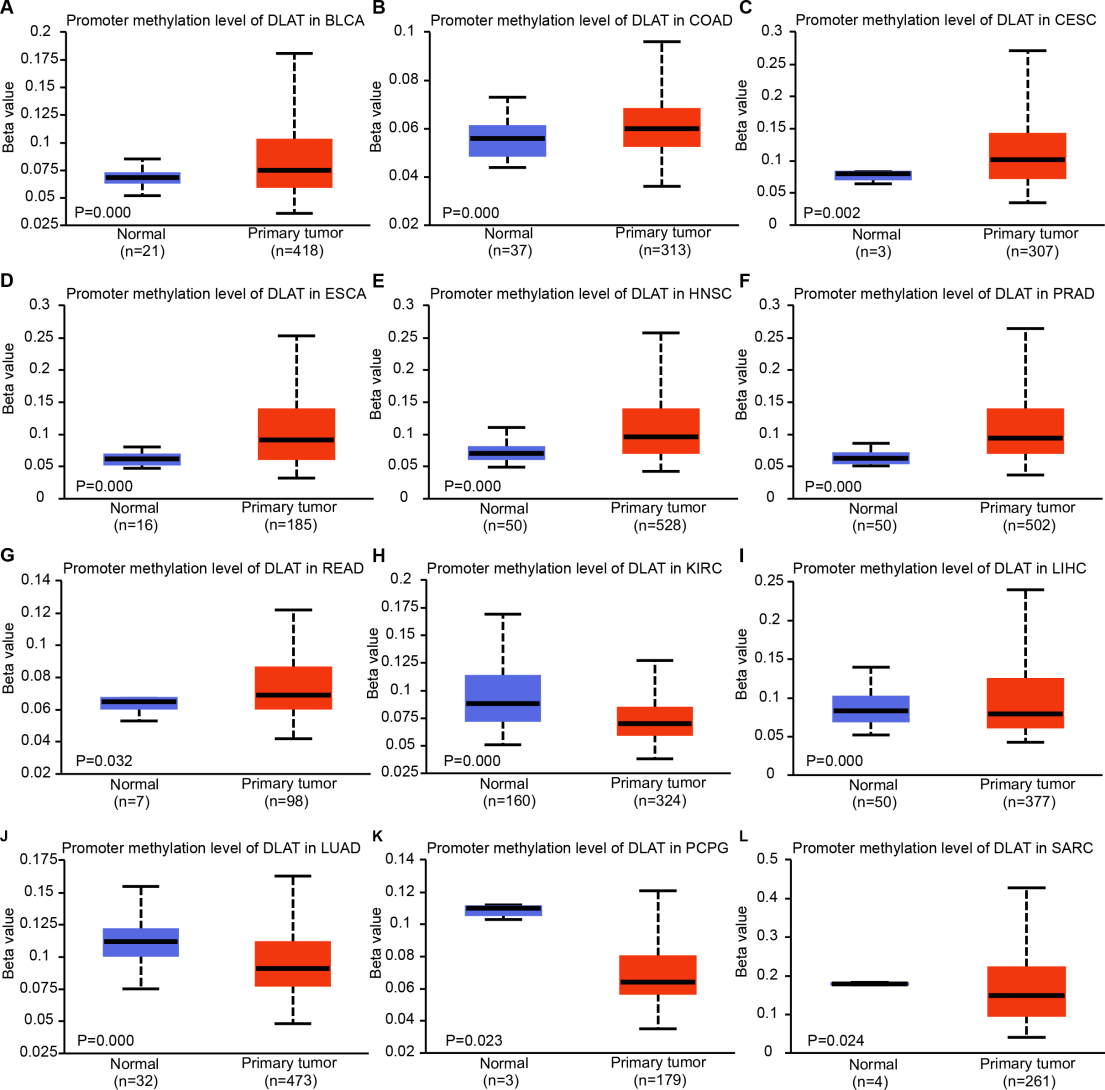
DNA methylation levels of DLAT in tumor analyzed using the UALCAN tool. (A) BLCA. (B) COAD. (C) CESC. (D) ESCA. (E) HNSC. (F) PRAD. (G) READ. (H) KIRC. (I) LIHC. (J) LUAD. (K) PCPG. (L) SARC.

Epigenetic modifications are mainly realized by DNA methylation, which has been proven to be a key contributor to the process of tumorigenesis and tumor progression (Abante et al., 2021; Chen et al., 2021a; Fatima et al., 2021; Zhang et al., 2021). Through comparing the methylation roles of DLAT in tumors and normal tissues, we found that the levels of DNA methylation were significantly increased in bladder urothelial carcinoma (BLCA), colon adenocarcinoma (COAD), cervical squamous cell carcinoma and endocervical adenocarcinoma (CESC), ESCA, HNSC, prostate adenocarcinoma (PRAD) and rectum adenocarcinoma (READ) (Figs. 2A–2G). On the contrary, we found relatively low DNA methylation levels of DLAT in KIRC, LIHC, LUAD, pheochromocytoma and paraganglioma (PCPG), and sarcoma (SARC) (Figs. 2H–2L). There were no significant differences in the DLAT methylation levels of tumors containing CHOL, glioblastoma multiforme (GBM), kidney renal papillary cell carcinoma (KIRP), LUSC, PAAD, STAD, thyroid carcinoma (THCA), thymoma (THYM) and uterine corpus endometrial carcinoma (UCEC) (Figs. S1A–S1I).

Using the cBioPortal tool, we analyzed the pan-cancer genetic alteration data of DLAT. In melanoma, the mutation frequency of DLAT genes is the highest among all cancers, with more than 4% higher than those frequencies of all other cancers. The “deep deletions” were the predominant type of mutation (Fig. S2A). In addition, Fig. S2 shows that the major variation type of DLAT is the missense mutation. The V242Wfs*5/E239G alteration, a truncating mutation in the Biotin_lipoyl domain, presented potential clinical significance in two cases of STAD, one case of UCEC, one case of invasive breast carcinoma (BRCA) and one case of COAD. Then, we investigated the effects of DLAT alterations on the survival rates of patients with different tumors. Compared with patients without DLAT alterations, the ESCA and LGG patients with DLAT alterations exhibited poor OS, progression-free survival (PFS), and disease-specific survival (DSS). In contrast, uterine carcinosarcoma (UCS) patients with DLAT alterations exhibited poor disease-free survival (DFS). Furthermore, the STAD patients with DLAT mutations showed favorable prognosis in PFS ( *P* = 0.0276), but not in OS, DSS, and DFS (Figs. S2K–S2N).

### Significant pan-cancer prognostic potential of DLAT

**Figure 3:**
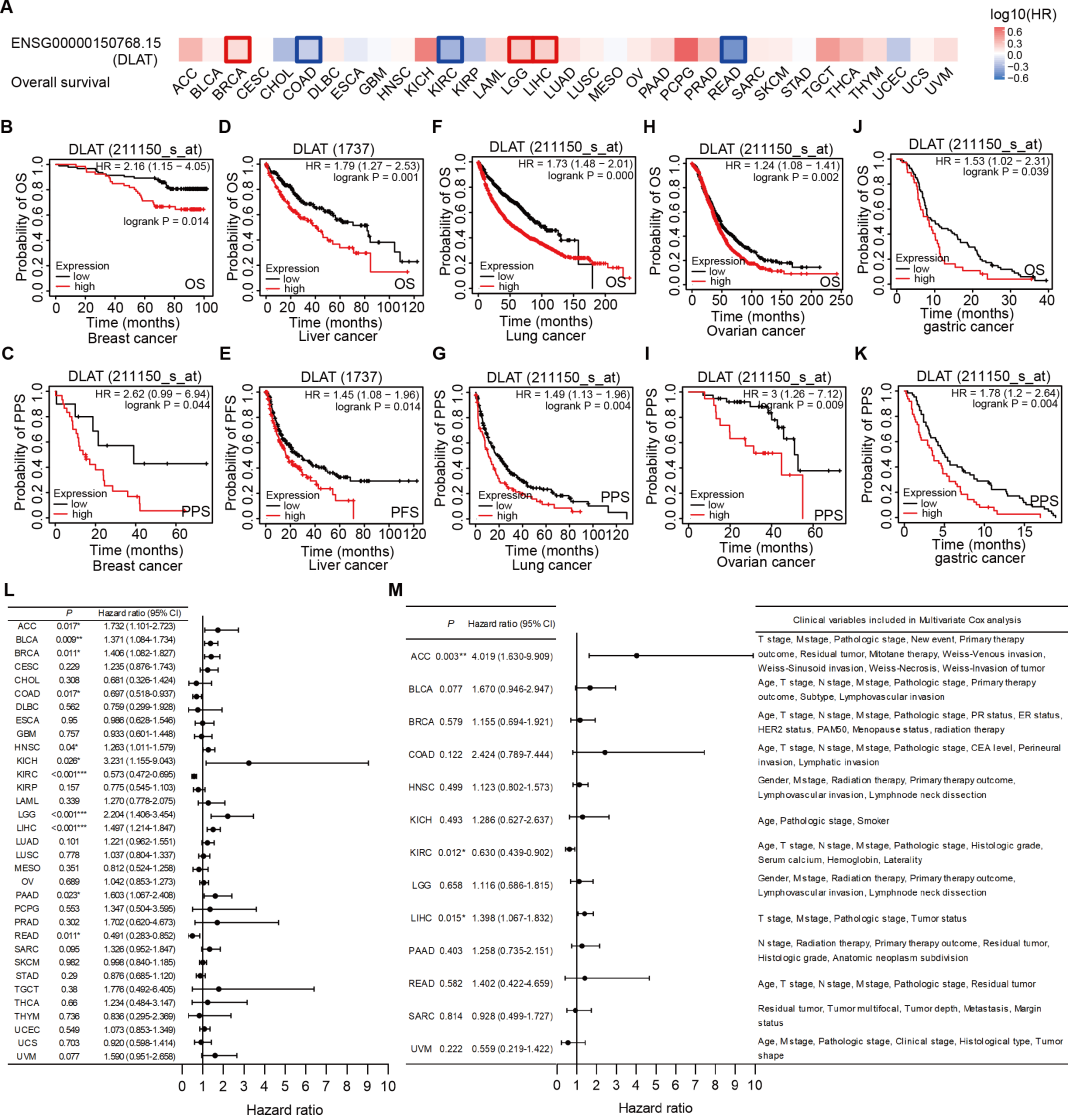
Correlation analysis of DLAT expression level and clinical prognosis in different tumors. (A) The survival maps of overall survival by GEPIA2. Tumor survival analyzed by Kaplan–Meier Plotter tool in breast cancer (B–C), liver cancer (D–E), lung cancer (F–J), ovarian cancer (H–I), and gastric cancer (J–K). Forest plots of associations between DLAT expression with overall survival in TCGA tumor types. (L) Univariate Cox regression analysis. (M) Multivariate Cox regression analysis adjusting for all available clinical variables. * *P* < 0.05; ** *P* < 0.01; *** *P* < 0.001.

The effects of DLAT levels on the clinical prognoses of different cancer patients were analyzed with the GEPIA2 website. High DLAT expression levels were observed among the BRCA (Logrank *P* = 0.038), LGG (Logrank *P* = 0.006), and LIHC (Logrank *P* = 0.007) patients and these levels were related to the poor OS of these patients. Meanwhile, those COAD (Logrank *P* = 0.037), KIRC (Logrank *P* = 0.000), and READ (Logrank *P* = 0.029) patients exhibited poorer OS than the patients mentioned above, which was related to their low levels of DLAT (Fig. 3A). In addition, with the Kaplan–Meier Plotter, we found that the patients of breast cancer, liver cancer, lung cancer, ovarian cancer, as well as gastric cancer had a relatively poor prognosis if they showed relatively high DLAT expression levels. Specifically, breast cancer patients with relatively high expressions of DLAT exhibited relatively poor OS, post-progression survival (PPS), relapse-free survival (RFS), and distant metastasis-free survival (DMFS) (Figs. 3B–3C, Figs. S3A–S3B). Meanwhile, liver cancer patients with relatively high expressions of DLAT showed relatively poor OS, PFS, RFS, and DSS (Figs. 3D–3E, Figs. S3D–S3E). Lung cancer patients with relatively high expressions of DLAT exhibited relatively poor OS, PPS, and first progression (FP) (Figs. 3F–3G, Fig. S3C). Furthermore, ovarian cancer patients with relatively high DLAT expressions presented relatively poor OS, PPS, and PFS (Figs. 3H–3I, Fig. S3F), while patients of gastric cancer with relatively high DLAT expressions showed relatively poor OS and PPS (Figs. 3J–3K).

In order to assess the prognostic significance of DLAT to the thirty-three tumor types in the TCGA, univariate and multivariate Cox regression analyses were conducted on the basis of the DLAT gene expressions and all available clinical variables, with OS being used as the dependent variable. The results of the univariate Cox regression analysis showed that DLAT expressions had an adverse effect on the OS of patients with adrenocortical carcinoma (ACC), BLCA, BRCA, HNSC, kidney chromophobe (KICH), LGG, LIHC and PAAD (Hazard ratio>1, *P* < 0.05). In contrast, DLAT expressions were related to a protective effect on the patients with COAD, KIRC, and READ (Hazard ratio<1, *P* < 0.05) (Fig. 3L). The variable criterion for the multivariate Cox regression analysis was that the *P* value of the univariate Cox regression should be less than 0.1. After all clinical variables were adjusted, the multivariate Cox regression analysis delivered the results which showed that the DLAT expressions had significantly adverse effects on patients with ACC (Hazard ratio =4.019; 95% CI [1.630–9.909]) and LIHC (Hazard ratio = 1.398; 95% CI [1.067–1.832]), and a significantly protective effect on patients with KIRC (Hazard ratio = 0.630; 95% CI [0.439–0.902]) (Fig. 3M).

### Effects of DLAT expressions on survival and chemotherapeutic resistance of LIHC patients

**Figure 4:**
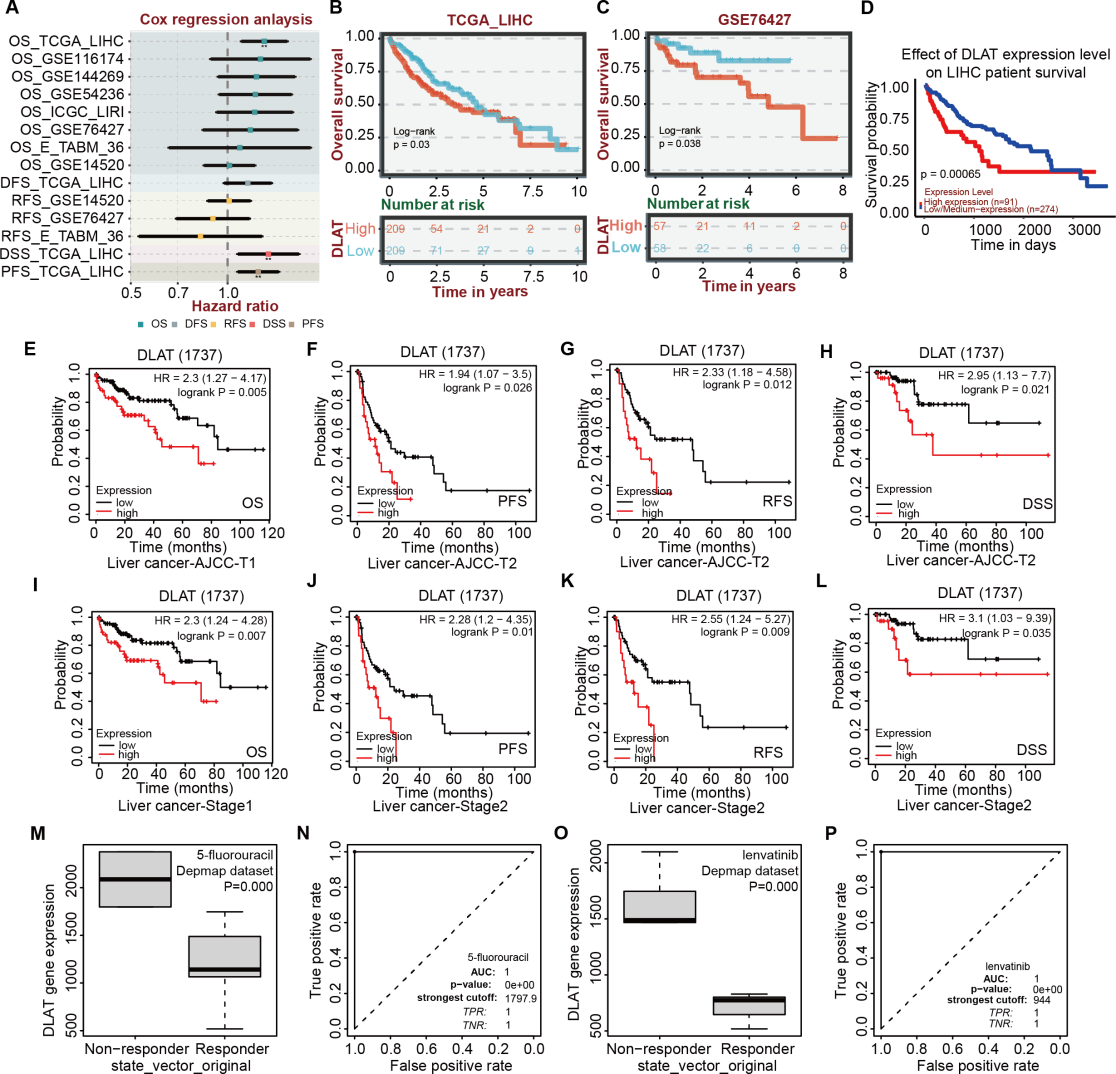
Correlation analysis of DLAT expression level, clinical prognosis and chemotherapeutic resistance in LIHC. (A) The cox regression analysis of survival by BEST. Overall survival of LIHC analyzed by BEST tool in TCGA dataset (B) and GES76427 (C). Effect of DLAT on LIHC survival basing on TCGA dataset by UALCAN tool (D). Kaplan–Meier analysis of DLAT expression and survival of subtypes in LIHC, including AJCC-T1 (E), AJCC-T2 (F–H), Stage1 (I) and Stage2 (J–L). Expression level of DLAT in non-responder group and responder group of 5-fluorouracil (M) and Lenvatinib (O) from Depmap dataset that analyzed by ROC plotter. AUC value of drug sensitivity prediction based on DLAT for 5-fluorouracil (N) and Lenvatinib (P). ** *P* < 0.01.

After verifying the different databases described above repeatedly, we tried to single out the tumor types that were consistent in mRNA and protein expressions of DLAT and exhibited a significant connection between DLAT level and prognosis. Previously, we obtained the result that the gene and protein levels of DLAT were upregulated in LIHC (Figs. 1A–1D). We also figured out that the DLAT expressions had a significant adverse effect on LIHC and that high expressions of DLAT were related to the poor prognoses of LIHC (Figs. 2A, 2D–2E and 2L–2M). Then, other online tools, including the BEST and the UALCAN, were utilized to verify the effect of DLAT expressions on the survival of the LIHC patients on the basis of the TCGA dataset and different GEO datasets. The results showed that relatively high expressions of DLAT were related to the relatively poor prognoses of the LIHC patients in the TCGA (Figs. 4A–4B and 4D) and the GSE76427 dataset (Fig. 4C). Since the multivariate Cox regression analysis used such clinical variables of LIHC as T stage, M stage, pathologic stage, and Tumor status, a prognosis analysis was conducted for sub-types of LIHC accordingly. The LIHC patients at the AJCC T1 and stage1 with relatively high DLAT expressions exhibited relatively poor OS (Figs. 4E and 4I). Furthermore, for patients of LIHC at the AJCC T2 and stage2, relatively high DLAT expressions were related to their relatively poor PFS, RFS, and DSS (Figs. 4F–4H and 4J–4L). The ROC plotter tool was used to analyze the correlation between DLAT gene expressions and the sensitivity of all chemotherapy drugs for LIHC. We found that in the Depmap dataset, the DLAT levels of patients in the non-responding groups to 5-Fluorouracil and Lenvatinib were significantly higher than those in the responding groups (5-Fluorouracil, Fig. 4M; Lenvatinib, Fig. 4O; both *P* = 0.000). And the predicted AUC values of drug sensitivity towards 5-Fluorouracil and Lenvatinib were 1 (Figs. 4N and 4P).

### The role of DLAT in regulating pro-survival autophagy in LIHC

**Figure 5:**
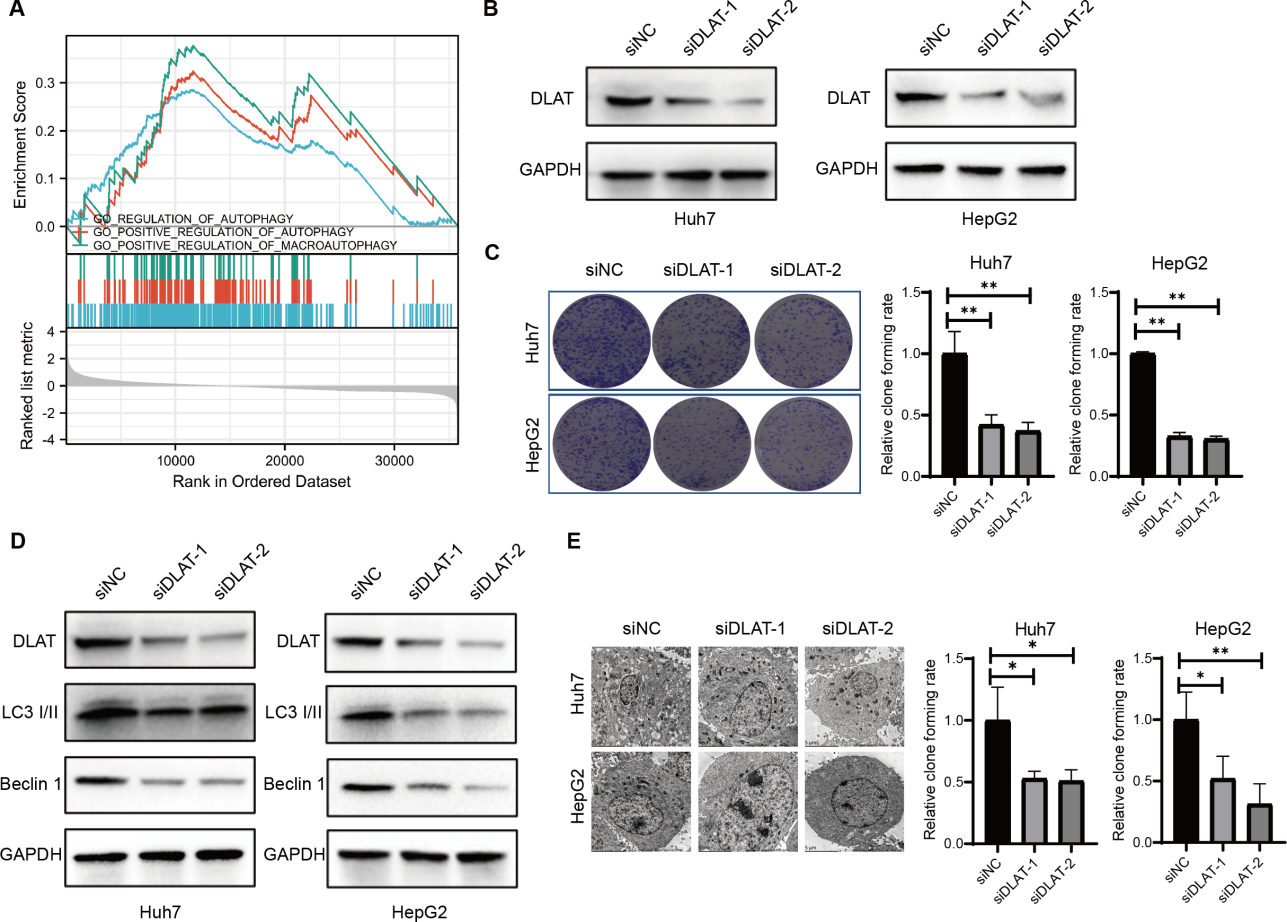
DLAT regulates autophagy in LIHC. (A) GSEA analysis on DLAT-related genes with autophagy. (B) After Huh7 and HepG2 cells were transfected with siNC or siDLAT, the protein level of DLAT protein was detected by western blot. (C) Huh7 and HepG2 cells transfected with siNC or siDLAT were analyzed in colony formation assays, and the *p* values were as follows: 0.0068, 0.0047, 0.000, 0.000. (D) Huh7 and HepG2 cells were transfected with siNC or siDLAT, following up with detection of DLAT, LC3 I/II and Beclin-1. (E) After Huh7 and HepG2 cells were transfected with siNC or siDLAT, the formation of autophagosome was observed under by TEM method, and the p values were as follows: 0.0418, 0.0404, 0.0452, 0.0131. * *P* < 0.05; ** *P* < 0.01.

Previous studies have found that DLAT colocalizes with autophagy markers LC3 and SQSTM1 among patients with primary biliary cirrhosis (PBC), and the autophagy induced in bile epithelial cells is related to the over-expression of DLAT (Sasaki et al., 2013; Zhang et al., 2020). Accordingly, we assessed the role of DLAT in mediating autophagy in LIHC cells. We conducted a single gene differential analysis to select the DLAT-related genes with which we performed the GSEA and verified that these DLAT-related genes might directly or indirectly affect autophagy (Fig. 5A). The DLAT broken-down LIHC cell lines (Huh7 and HepG2) were cultured with two siRNAs (Fig. 5B). A colony formation analysis revealed that the breaking down of DLAT significantly inhibited the colony formation of Huh7 and HepG2 cells (Fig. 5C). Furthermore, protein markers such as LC3 I/II and Beclin-1 involved in the autophagosome formation were determined *in vitro*. Inhibiting DLAT with siRNA other than with siNC in Huh7 and HepG2 cells could decrease the expressions of LC3 I/II and Beclin-1 (Fig. 5D). Consistently, the TEM analysis showed that the breaking down of DLAT in Huh7 and HepG2 cells reduced the number of autophagosomes significantly (*P* < 0.05; Fig. 5E). In general, all these analysis results showed that the breaking down of DLAT can inhibit the pro-survival autophagy in LIHC cells.

### The effects of DLAT on Tregs infiltration in LIHC

**Figure 6:**
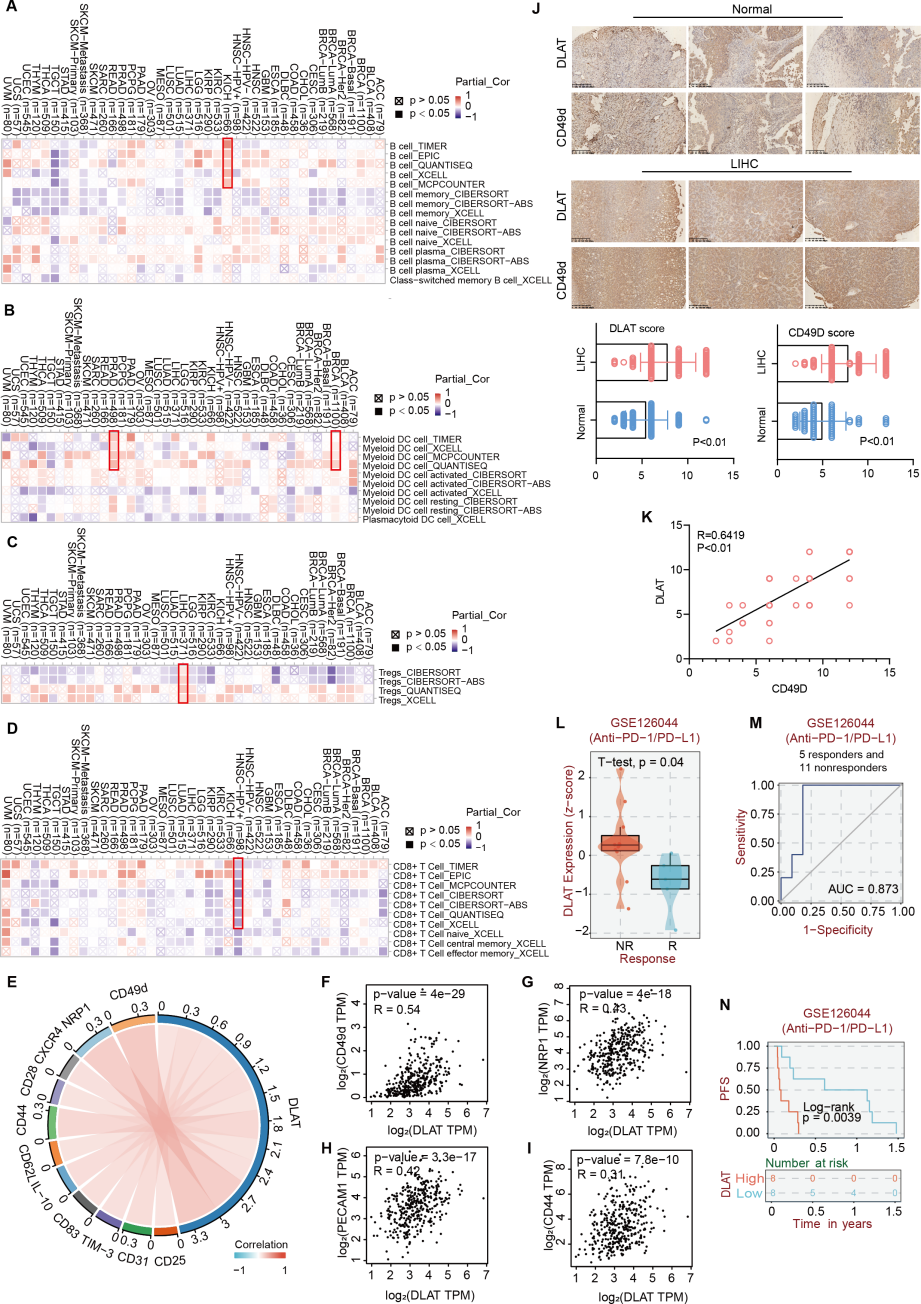
The effects of DLAT on Tregs in LIHC. Correlation analysis between DLAT and B cell (A), DC cell (B), Tregs (C) and CD8+ T cell (D) across different cancers in TCGA. (E) Relationships in DLAT with typical markers of Tregs in LIHC from TCGA dataset were analyzed by Xiantao. (F–I) Correlation analyses between DLAT and the Tregs markers CD31, CD44, NRP1 and CD49d by GEPIA2. (J) IHC analysis in expression of DLAT and CD49d in LIHC and normal tissues. (K) The staining intensity of CD49d and DLAT. Correlations of differential expression of DLAT with response (L–M) and prognosis (N) of immunotherapy were analyzed by BEST.

Studies have revealed that DLAT plays an immunomodulatory role in some autoimmune diseases. Thus, some studies have analyzed the possibility of DLAT affecting the occurrences and development of tumors by regulating immune infiltration (Wang et al., 2016). Multiple algorithms from the TIMER2.0 website were applied to analyze the roles of DLAT in regulating the immune infiltration of all TCGA tumors. The analysis results showed that there were positive correlations between DLAT and B cells in KICH (spearman’s R: 0.252−0.548, *P* < 0.05, Fig. 6A) and between DLAT and DC cells in BRCA (spearman’s R: 0.025−0.209, *P* < 0.05) and PRAD (spearman’s R: 0.148−0.328, *P* < 0.05) (Fig. 6B). In addition, there was also a positive correlation between DLAT and Tregs in LIHC (spearman’s R: 0.114−0.267, Fig. 6C). However, DLAT was negatively correlated with the infiltration of CD8+ T cell in HPV+ HNSC (spearman’s R: −0.483-(−0.233), Fig. 6D). No significant correlation was observed between DLAT and other immune cells (Figs. S4A–S4E). Furthermore, when analyzing the relationships between immunotherapy responses and DLAT expressions of various tumors, we found that among all the patients in anti-PD-1 / PD-L1 treatment of GSE126044, the patients in the non-responding group showed relatively high DLAT expressions (Fig. 6L), which were related to the relatively poor PFS (Fig. 6N). From the ROC curve, it can be seen that the DLAT-based AUC value of immunotherapy response prediction is 0.873 (Fig. 6M).

Using the Xiantao tool and GEPIA2 tool, we conducted an analysis based on the LIHC dataset in TCGA to analyze the relationships between DLAT and such classical phenotypic markers of Tregs as CD25, CD31, TIM-3, CD83, IL-10, CD62L, CD44, CD28, CXCR4, NRP1, and CD49d (Doebbeler et al., 2018; Peterson, 2012; Sakaguchi et al., 2020; Scheinecker, Goschl & Bonelli, 2020) (Fig. 6E). The analysis results showed significant positive correlations between the Tregs markers of CD31, CD44, NRP1, and CD49d and DLAT (spearman’s *R* > 0.3, *P* < 0.05) in LIHC (Figs. 6F–6I). Furthermore, the IHC staining results of tissue micro-array specimens showed that the expression intensities of DLAT and CD49d in LIHC were significantly higher than those in normal specimens (Fig. 6J). In terms of staining intensity, there was a significant positive correlation between the typical Tregs marker of CD49d and DLAT (Pearson’s *R* = 0.6419, *P* = 0.000) (Fig. 6K).

## Discussion

With the molecular mechanisms and biological functions of DLAT in tumorigenesis not yet fully revealed, our team initiated the pan-cancer analysis to find a solution. Our results showed that compared with their adjacent normal tissues, LIHC, LUAD, LUSC, and STAD had higher mRNA expressions and IHC staining of DLAT, while HNSC and KIRC had lower ones. It was also found that LIHC, LUAD, LUSC, and STAD patients with relatively high DLAT expressions and KIRC patients with relatively low DLAT expressions tended to exhibit relatively poor clinical prognoses. These results indicated that DLAT could be used as a prognostic marker for LIHC, LUAD, LUSC, STAD, and KIRC. Especially, the mRNA and protein expressions of DLAT in LIHC were higher than normal levels. Also, both Kaplan–Meier analysis and multivariate Cox regression analysis revealed that high levels of DLAT were closely related to poor prognoses of LIHC.

In this study, we also observed that DLAT expressions were positively correlated with B cells in KICH, DC cells in BRCA and PRAD, and Tregs in LIHC. In contrast, they were negatively correlated with CD8+ T cells in HPV+ HNSC. Previous studies have confirmed that DLAT plays a role in autoimmune diseases, such as PBC, a liver-specific autoimmune disease. It has been confirmed that the amounts of DLAT-specific CD4+ T cells and CD8+ T cells in liver tissues of PBC patients are nearly 100-fold and 10-fold higher than those in the peripheral blood, respectively (Wang et al., 2016). The oxoglutarate dehydrogenase complex, which is a major mitochondrial autoantigen, together with DLAT, brings about the enhanced responses of CD4+ and CD8+ T cells in PBC (Berg, 2011; Xu, Shen & Ran, 2020). La Rocca et al. have observed reduced expressions of DLAT during T cell activation among patients with multiple sclerosis, another autoimmune disease (La Rocca et al., 2017). Some current studies show that there exists an immune-stimulating tumor micro-environment among tumor cells with high glycolysis levels (Deng et al., 2021; Jiang et al., 2021; Yang et al., 2021). These results preliminarily indicate the potential involvement of DLAT in the immune regulation of cancers.

Huang et al. (2019) have found that DLAT can promote the metabolism and biogenesis of LIHC cells through MET kinase-mediated phosphorylation. Similarly, our survival analysis based on TCGA revealed negative correlations between DLAT and OS, DSS, PFS, and RFS in LIHC. Also, we discovered a possible relationship between DLAT expressions and the sensitivities to 5-Fluorouracil and Lenvatinib among LIHC patients. This finding is consistent with the results drawn by Huang et al. (2019) in their study on HGF-MET targeted drug resistance, which proposed that DLAT may be a new target to overcome the chemotherapeutic resistance in LIHC. Moreover, we found that DLAT expressions were positively correlated with patients’ PD-L1 levels and high sensitivities to PD-L1 antibodies, which indicated that DLAT might play an essential role in immunotherapy synergism (Bai et al., 2022; Zhang et al., 2022a). Accordingly, the immune infiltration analysis revealed a positive correlation between DLAT and Tregs in LIHC and identified the significant positive correlations between DLAT and typical phenotype markers of Tregs, such as CD49d. Also, with IHC staining, strong staining intensities of DLAT and CD49d in LIHC were observed, and their correlation was identified. Previous studies have confirmed the correlations between DLAT and enhanced responses of CD4+ and CD8+ T liver cells of PBC patients (Shimoda et al., 1998; Wang et al., 2016). It has also been confirmed that Tregs can promote tumor progression and invasion, resulting in poor prognosis and resistance to sorafenib in LIHC. Also, removing Tregs has become a possible immunotherapy to decrease postoperative recurrence and improve survival (Gao et al., 2007; Zhou et al., 2016). Therefore, with our results combined with the results of previous studies, it is proposed that DLAT is highly expressed in LIHC, leading to increased immune infiltration of Tregs, which may inhibit the responses of CD4+ and CD8+ T cells. Then, tumorigenesis and tumor invasion are ultimately promoted with the anti-cancer immunity inhibited.

Moreover, our experiments verified that DLAT promoted cell growth in LIHC by targeting autophagy. We discovered that the down-regulation of DLAT in different LIHC cells led to decreases in LC3 I/II and Beclin-1 and in colony formation and autophagosomes. Autophagy is an important process to ensure genomic integrity and cell stability, acting as a double-edged sword determining the survival and death of tumor cells under different tumor development stages (Camuzard et al., 2020; Das, Mandal & Kogel, 2018). By referring to the few studies so far on the relationship between DLAT and autophagy, we know that enhanced DLAT expressions may induce autophagy in bile epithelial cells (Sasaki et al., 2013; Xu, Shen & Ran, 2020). One study revealed that MET-mediated phosphorylation inhibits the activity of PDC, including DLAT, and promotes the metabolism and biogenesis of LIHC cells. In contrast, dephosphorylated MET promotes LIHC cell survival by inducing autophagy (Huang et al., 2019). Additionally, through constructing a Treg-restricted autophagy deficiency mouse model, it was found that the deletion of essential autophagic genes would result in the loss of Tregs and enhance anti-tumor immunity (Wei et al., 2016; Zhang et al., 2019). In terms of mechanism, autophagy ensures the functional integrity of Tregs by inhibiting excessive apoptosis and enhancing metabolic homeostasis (Zhang et al., 2019). The autophagy defects lead to the up-regulation of mTORC1 and c-Myc and the enhancement of glycolysis, thus resulting in functional defects of Tregs (Wei et al., 2016). Our study showed that DLAT could promote the survival of LIHC cells by affecting the autophagy and immune infiltration of Tregs, indicating that targeting DLAT may be a potential approach to inhibit the growth of LIHC. With the consideration that there exists a close relationship between autophagy and Tregs, the regulation mechanisms of DLAT on Tregs through autophagy in LIHC should be further clarified. Recent studies applying the copper death signaling pathway have also pointed out that DLAT is closely related to copper-induced cell death and that DLAT may be involved in tumorigenesis and development (Tsvetkov et al., 2022).

This study has provided a reference for comprehensively understanding the pan-cancer expressions and roles of DLAT. Specifically, this study has revealed that DLAT may promote the tumor progression of LIHC by enhancing pro-survival autophagy and immune infiltration of Tregs and may participate in the resistance of LIHC to targeted drugs. These results have provided a meaningful perspective for treating LIHC and paved a new way to overcome the serious clinical problem of drug resistance in advanced LIHC. However, this study primarily conducted bioinformatics analyses based on pan-cancer data. The molecular mechanisms of DLAT regulating autophagy in LIHC and the relationship between autophagy and Tregs are yet to be clarified. Also, whether DLAT participates in the resistance of LIHC to Lenvatinib should be verified with *in vivo* and *in vitro* experiments, which will be further discussed in the follow-up study.

## Conclusions

In conclusion, our study has revealed that relatively high DLAT expressions among LIHC, LUAD, LUSC, and STAD patients and relatively low DLAT expressions among KIRC patients are related to these patients’ poor prognoses. Especially, DLAT could promote the growth of LIHC cells by targeting autophagy and lead to increased immune invasion of Tregs, thus inhibiting the responses of CD4+ and CD8+T cells and ultimately promoting the occurrence of LIHC by inhibiting anti-tumor immunity. Our pan-cancer analysis on DLAT has enhanced the understanding of the expressions, prognostic potential, immunological functions, and possible mechanisms of DLAT in various cancers. In particular, our analysis has pointed out new research directions for tumor immunity and the therapeutic target of LIHC. However, most results of this study were obtained through bioinformatics analysis. In order to further verify these results, a series of experiments needs to be performed.

##  Supplemental Information

10.7717/peerj.15019/supp-1 Figure S1 DNA methylation levels of DLAT in tumor and normal tissues of TCGA were analyzed by UALCAN tool (A) CHOL. (B) GBM. (C) KIRP. (D) LUSC. (E) PAAD. (F) STAD. (G) THCA. (H) THYM. (I) UCEC.

10.7717/peerj.15019/supp-2 Figure S2 Mutation characteristics of DLAT in different tumors of TCGA which analyzed by the cBioPortal tool The alteration type (A) and site information (B) were presented. Analysis of survival differences between cancer patients in DLAT-altered group and DLAT-unaltered group of ESCA (C-F), LGG (G-J), STAD (K-N) and UCS (O-R).

10.7717/peerj.15019/supp-3 Figure S3 Relationships between DLAT expression and clinical prognosis in different tumors Associations between DLAT expression and multiple prognostic markers were analyzed by Kaplan-Meier Plotter tool in breast cancer (A-B), lung cancer (C), liver cancer (D-E) and ovarian cancer (F).

10.7717/peerj.15019/supp-4 Figure S4 The roles of DLAT in the regulation of immune infiltration cells Correlation analysis between DLAT expression and cancer associated fibroblast (A) and NK cell (B), macrophage (C), monocyte (D), and neutrophil (E) across different cancers in TCGA.

10.7717/peerj.15019/supp-5 Supplemental Information 5 Raw data for Figure 1 (A-J)

10.7717/peerj.15019/supp-6 Supplemental Information 6 Raw data for Figure 2 (A-L)

10.7717/peerj.15019/supp-7 Supplemental Information 7 Raw data for Figure 3 (A-M)

10.7717/peerj.15019/supp-8 Supplemental Information 8 Raw data of Figure 4 (A-L)

10.7717/peerj.15019/supp-9 Supplemental Information 9 Raw data of Figure 5 (A-E)

10.7717/peerj.15019/supp-10 Supplemental Information 10 Raw data for Figure 6 (A-N)

10.7717/peerj.15019/supp-11 Supplemental Information 11 Raw data for Figures S1-S4

## Additional Information and Declarations

### Data Availability

The following information was supplied regarding data availability: The raw data are available in the Supplementary Files.
