# Supplementary figures and images for: Roles of cuproptosis-related gene DLAT in various cancers: a bioinformatic analysis and preliminary verification on pro-survival autophagy
Source: PeerJ. 2023 Mar 17;11:e15019. doi: 10.7717/peerj.15019 (PMC10026716; doi:10.7717/peerj.15019)

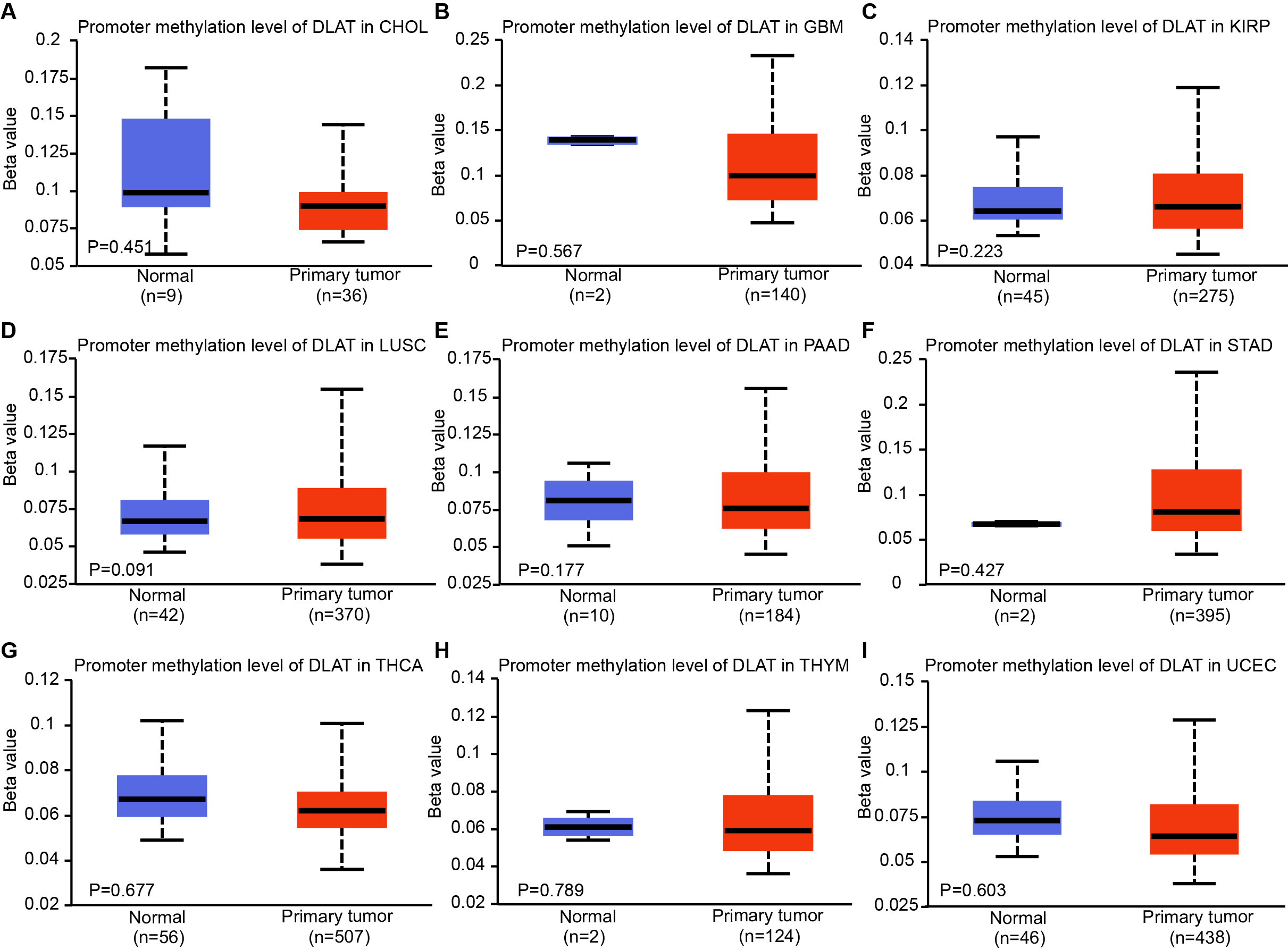

Supplement: Figure S1 — (A) CHOL. (B) GBM. (C) KIRP. (D) LUSC. (E) PAAD. (F) STAD. (G) THCA. (H) THYM. (I) UCEC. [file peerj-11-15019-s001.png]

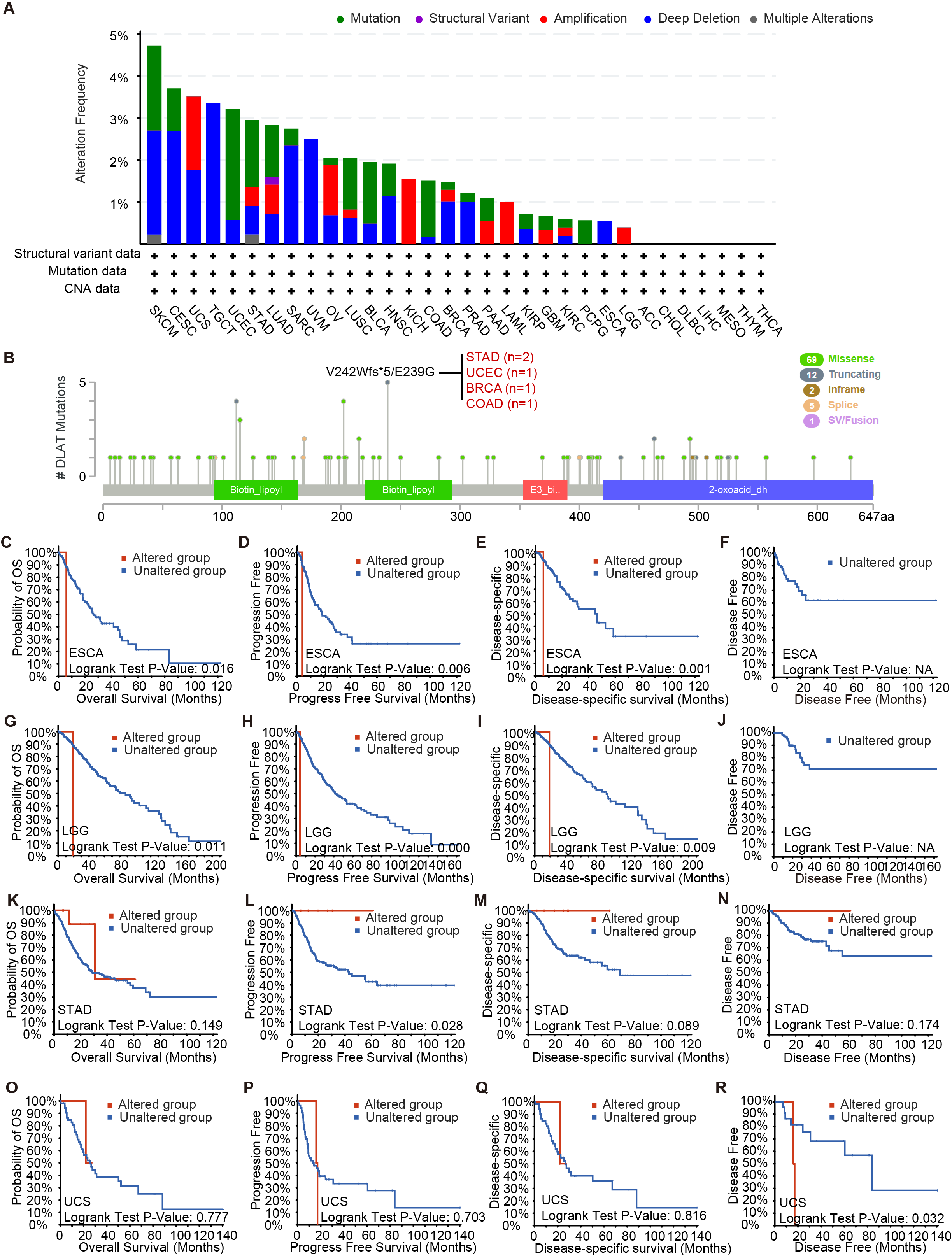

Supplement: Figure S2 — The alteration type (A) and site information (B) were presented. Analysis of survival differences between cancer patients in DLAT-altered group and DLAT-unaltered group of ESCA (C-F), LGG (G-J), STAD (K-N) and UCS (O-R). [file peerj-11-15019-s002.png]

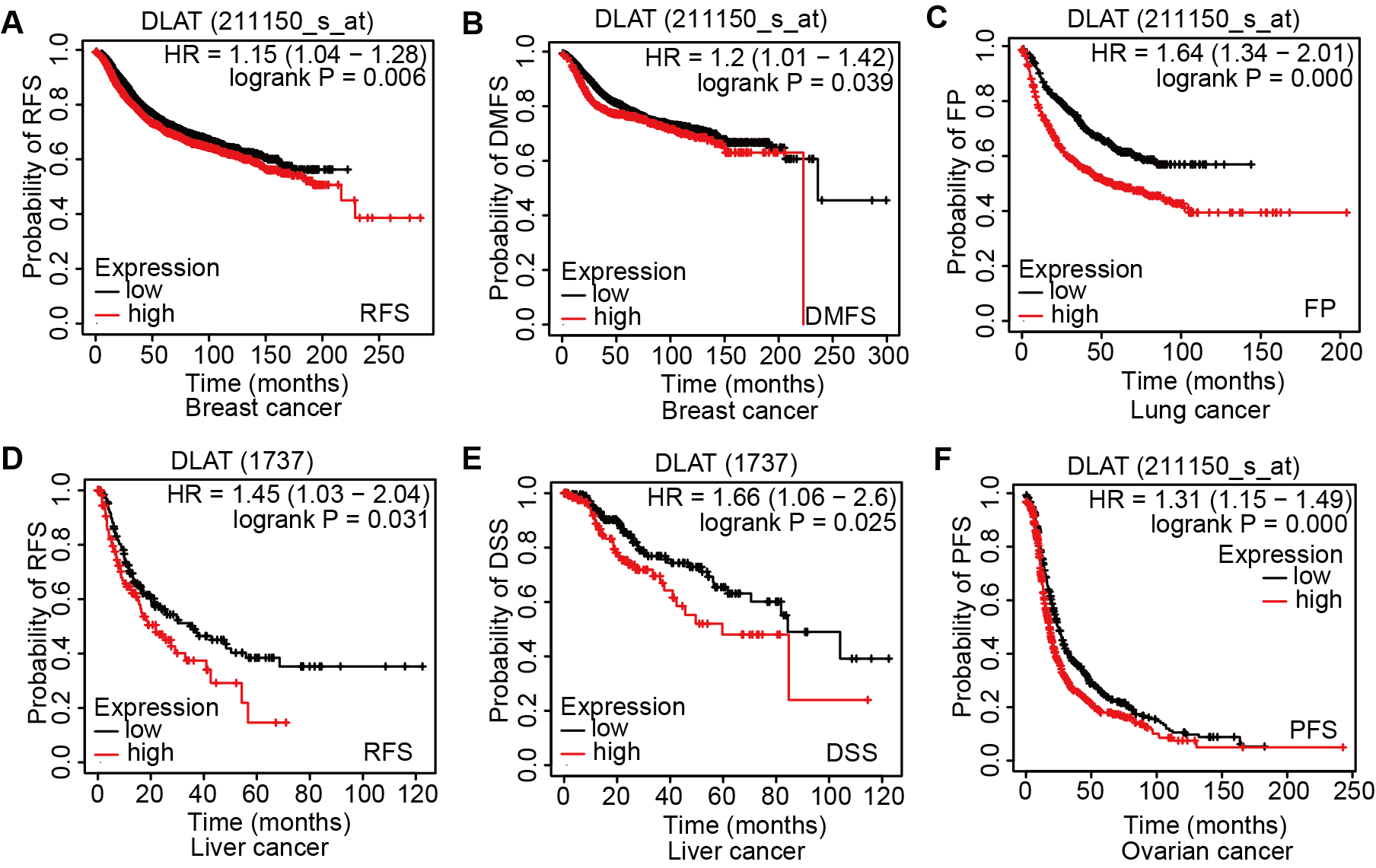

Supplement: Figure S3 — Associations between DLAT expression and multiple prognostic markers were analyzed by Kaplan-Meier Plotter tool in breast cancer (A-B), lung cancer (C), liver cancer (D-E) and ovarian cancer (F). [file peerj-11-15019-s003.png]

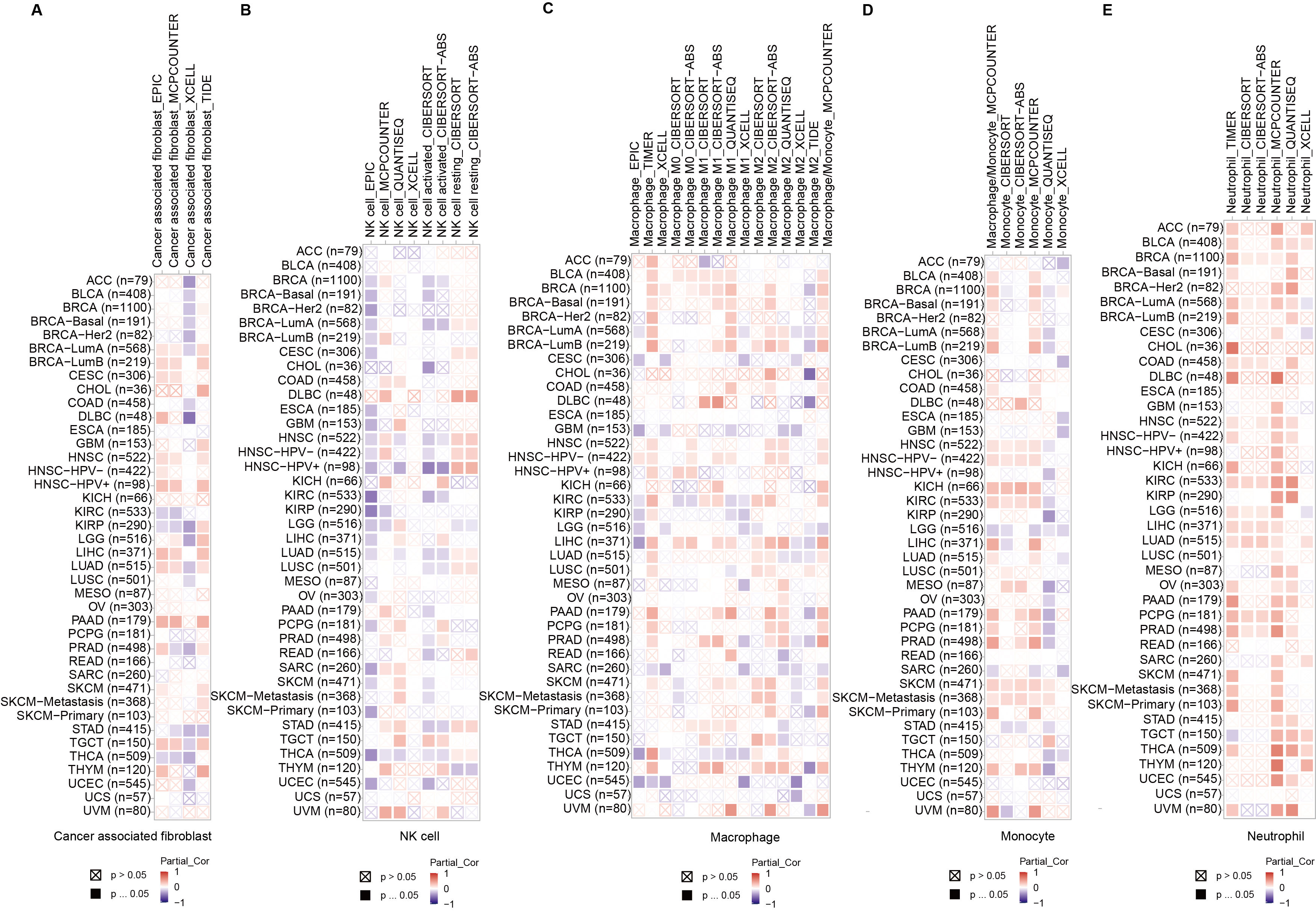

Supplement: Figure S4 — Correlation analysis between DLAT expression and cancer associated fibroblast (A) and NK cell (B), macrophage (C), monocyte (D), and neutrophil (E) across different cancers in TCGA. [file peerj-11-15019-s004.png]
